# Classification of Corn Diseases from Leaf Images Using Deep Transfer Learning

**DOI:** 10.3390/plants11202668

**Published:** 2022-10-11

**Authors:** Mohammad Fraiwan, Esraa Faouri, Natheer Khasawneh

**Affiliations:** 1 Department of Computer Engineering, Jordan University of Science and Technology, Irbid 22110, Jordan; 2 Department of Software Engineering, Jordan University of Science and Technology, Irbid 22110, Jordan

**Keywords:** corn, maze, leaf spot, rust, leaf blight, deep learning, artificial intelligence

## Abstract

Corn is a mass-produced agricultural product that plays a major role in the food chain and many agricultural products in addition to biofuels. Furthermore, households in poor countries may depend on small-scale corn cultivation for their basic needs. However, corn crops are vulnerable to diseases, which greatly affects farming yields. Moreover, extreme weather conditions and unseasonable temperatures can accelerate the spread of diseases. The pervasiveness and ubiquity of technology have allowed for the deployment of technological innovations in many areas. Particularly, applications powered by artificial intelligence algorithms have established themselves in many disciplines relating to image, signal, and sound recognition. In this work, we target the application of deep transfer learning in the classification of three corn diseases (i.e., Cercospora leaf spot, common rust, and northern leaf blight) in addition to the healthy plants. Using corn leaf image as input and convolutional neural networks models, no preprocessing or explicit feature extraction was required. Transfer learning using well-established and well-designed deep learning models was performed and extensively evaluated using multiple scenarios for splitting the data. In addition, the experiments were repeated 10 times to account for variability in picking random choices. The four classes were discerned with a mean accuracy of 98.6%. This and the other performance metrics exhibit values that make it feasible to build and deploy applications that can aid farmers and plant pathologists to promptly and accurately perform disease identification and apply the correct remedies.

## 1. Introduction

Plant diseases are one of the major factors in the destruction of agricultural crops, with an estimated global average yield loss of 2000 billion US dollars each year [1,2]. Plants are adversely affected by infection, which may be fungal, viral, or bacterial. These diseases attack leaves, fruits, or branches and may appear in the form of changes in color, shape, or edges. Thus, it is imperative to investigate possible fast detection, control, and treatment options. Traditional naked eye inspection is the first line of defense toward disease diagnosis [3], but it is prone to errors [4,5]. Furthermore, farmers can employ the wrong type of treatment method or chemical [6]. It generally needs a plant pathologist to analyze and discover the type of infection, which may be costly, not prompt enough, or unavailable [7,8].

Corn, or maze, is a worldwide mass-produced agricultural crop. In addition to being consumed directly as food, it forms the basis for many other products such as cooking oil, starch, flour, sugar, biofuel, alcohol, and animal feed. Corn grows in varying environmental conditions and it is considered one of the most important crops characterized by high genetic diversity and production potential, along with rice and wheat [9]. The global corn production reached 1.15 billion tons in 2020 [10]. It is naturally exposed to many diseases that can attack various parts of the plant including the leaves, trunk, and fruit at all growth stages. This has a direct impact on the yield of corn harvest and consequently can lead to great financial loss. On a global scale, depleted agricultural production of staple crops such as corn can lead to food shortages, hunger, or even famine [11]. Diseases that infect corn leaves during the growth process are the most dangerous of these maladies. Hence, this work considers three common leaf diseases [12]: Cercospora leaf spot, common rust, and northern leaf blight.

Cercospora leaf spot is caused by a two types of fungi, Cercospora zeae-maydis and Cercospora zeina, which inflect damage to the color and shape of the leaves and cause reduction in productivity. These fungi live on the soil surface and need a moist and warm environment and cause necrotic lesions of brown to grey color. Common treatments include the use of appropriate agent, but they must be conducted before the start of grain formation [13,14]. Northern Leaf blight is another type of fungal infection caused by the Exserohilum turcicum fungus. It can lead to great loss in agricultural crops if it attacks during the growth and reproduction stages. Moreover, it is exacerbated by certain weather conditions. The disease appears on the leaves in the form of dark brown spots with a straight rectangular shape. It is treated by the right corresponding chemical agent [15,16]. Common rust is caused by a fungus called Puccinia sorghi, and appears as dark blisters of reddish-brown color on both the upper and lower surfaces of corn leaves. It is treated with the help of the appropriate chemicals [17,18,19].

Technological advances have powered many agricultural innovations. More specifically, artificial intelligence (AI) and deep learning algorithms can use plant images to drive decision-making applications. Deep learning is based upon neural networks that are comprised of a number of layers far greater than the input, output and hidden layers. Convolutional neural networks (CNNs) are a subclass of deep learning networks that are concerned with finding features and relationships in images through a sequence of convolution, pooling and rectified linear unit (ReLU) layers that feed to a fully connected layer. This layer combines the various features discovered by the former layers. CNNs have been shown to be effective in discerning features regardless of many changes that can affect the input images [20]. In designing CNNs-based systems, researchers can build the network structure from scratch. Alternatively, existing reliable and well-established models can be reused and their learned knowledge can be transferred to other applications in what is known as transfer learning. Deep transfer learning adapts existing models with retraining partially or fully in a manner that fits the new application. It has the advantages of detecting generic features (e.g., colors, borders) with earlier layers and customizing later layers for specific applications.

Recently, several research works in AI-based corn disease detection and classification were conducted. Padilla et al. [21] designed a system using Raspberry Pi to capture and process corn leaf images and classify the type of disease exhibited by those leaves. They used CNN implemented using the multiprocessing programing library OpenMP. However, the paper does not include a description of the CNN architecture. The highest accuracy that they reported was 93% for the classification of leaf blight. However, the rust and leaf spot diseases were detected with an 89% accuracy. Panigrahi et al. [22] designed a CNN architecture that is nine layers deep. They reported an accuracy of 98.78% and an F1-score of 98.7%. However, little details are provided about the experimental setup in terms of how randomness in splitting the data is managed and how to guard against lucky choices. Recently, Amin et al. [23] fused features from two CNN models, EfficientNetB0, and DenseNet121, to produce a more representative feature map and hence superior performance. They experimented with multiple fusion techniques and compared the performance of their approach, which produced a superior accuracy of 98.56%, to that of the ResNet152 and Inceptionv3 models. However, the reported performance may have been skewed by the authors’ choice to increase the size of the dataset by augmentation. Subramanian et al. [10] used transfer learning and employed four CNN models: ResNet50, VGG16, Inceptionv3, and Xception. They tweaked the models’ hyperparameters and measured the accuracy for individual disease classes, which ranged from 83.92% to 99.9%. Xu et al. [24] modified an AlexNet CNN by adding a new convolution layer and an inception module. They compared their model with transfer learning based on VGGNet-16, DenseNet, ResNet-50 and AlexNet. Their modified model achieved a 93.28% accuracy.

The research landscape on the use of AI in agriculture and specifically in disease identification is ripe for more innovation and further confirmative studies. The work in this paper evaluates transfer learning using a wide range of CNN models to classify corn diseases. Instead of designing a CNN model from scratch, the work employs efficient well-known models that had undergone extensive evaluation to gain their place in the literature. Furthermore, the approach makes deployment and implementation easier by not requiring explicit feature extraction or elaborate image preprocessing. The contributions of this paper are as follows:

- Using leaf images as input, develop deep transfer learning models for the classification of corn diseases. Three such diseases were considered in this study: Cercospora leaf spot, common rust, and northern leaf blight. In addition, a fourth healthy class was included.
- Implement transfer learning with ten deep convolutional neural network models for the classification of leaf images into four classes.
- Evaluate the performance of the various models using multiple metrics that cover many aspects of the detection and classification capabilities. Moreover, the training and valuations times were reported. The results show that wrapping such models in mobile and smartphone devices can aid farmers to quickly and correctly identify diseases.

The remainder of this paper is organized as follows. Section 2 describes the dataset, deep learning models, the experimental setup and performance metrics. The results are described and discussed in Section 3. In Section 4, we present our conclusions.

## 2. Materials and Methods

**Figure 1:**
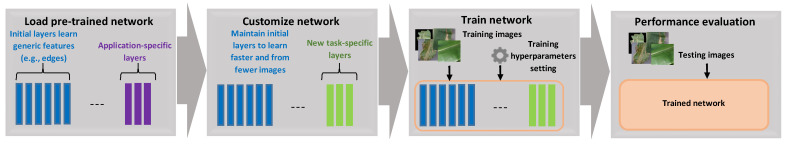
The general steps taken to build the corn disease classification model.

Given a dataset of corn leaf images representing a number of diseases and a healthy state, the goal of this work is to classify leaf images into the correct state. The methods in this work follow the transfer learning approach, where generically pre-trained deep learning models are customized to the new application (e.g., number of classes, and type of output) and re-trained on the new data. In transfer learning, initial (i.e., early) layers detect common low-level features (e.g., colors, edges, blobs) and later layers learn domain-specific features. Transfer learning utilizes this knowledge, and enables faster training in comparison to randomly setting initial weights. In addition, it makes it possible to learn from smaller number of images. Deep transfer learning has been shown to be useful and effective in a diverse set of applications from many disciplines [25]. The general steps followed in this work are shown in Figure 1. In the next few subsections, we go through each step in detail.

### 2.1. Dataset

**Figure 2:**
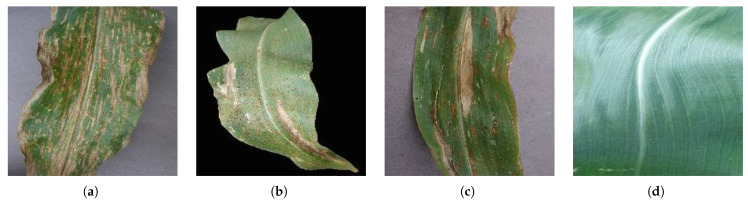
Sample images from the three disease classes and the healthy one: (**a**) Cercospora leaf spot. (**b**) Common rust. (**c**) Northern leaf blight. (**d**) Healthy.

The dataset is comprised of 3852 corn leaf images divided into four folders each representing one class of four possible disease states, as follows: 513 Cercospora leaf spot, 1192 common rust, 985 northern leaf blight, and 1162 healthy [26]. The leaf images are in JPEG format with a size of 256 × 256 pixels. Each file contains an image of one leaf only. Samples of the images of the three diseases and healthy leaves are shown in Figure 2.

### 2.2. Convolutional Neural Network Models

The CNN architecture is more elaborate than the traditional multilayer perceptron (MLP) networks (i.e., artificial neural networks (ANN)). MLP networks generally rely upon explicitly extracted numerical features performed through signal/image processing techniques. However, in themselves, they are unable to capture correlations and relative positions between various parts of the image. In other words, minor changes in scale, orientation, or positions can significantly degrade their performance. On the other hand, deep learning CNN involves building a network of layers that apply filters of various scales on the image to extract features. Subsequent layers reduce the dimensionality of the data for a lower overhead, aggregate information from the various feature maps, and improve the learning process by the introduction on non-linearity. The neural networks are comprised of three layers: input, output, and hidden. On the other hand, deep learning involves a far greater number of layers, which enables the capturing of input features and details at various scales. CNNs have been shown throughout the literature to be exceptionally well at handling image-based classification problems [20].

A plethora of custom CNN models have been proposed in the literature to handle specific applications. Furthermore, technology companies (e.g., Google Inc., Mountain View, CA, USA) have made some complex well-designed generic deep learning CNN models. These models were trained using large datasets (e.g., ImageNet [27]) to classify 1000 different image categories. Building on the credibility and power of these models, it is possible to utilize their architecture and learned parameters in a process called transfer learning. This approach offers the benefit of the network structure design and the learned model values. Retraining later parts of their network will employ the network structure in learning new task-related features and benefit from earlier layers in detecting non-application specific features (e.g., colors, boundaries) [28,29]. In this research, ten public CNN models were utilized through transfer learning to classify corn diseases based on leaf images. These were: DarkNet-53 [30], DenseNet-201 [31], GoogLeNet [32], Inceptionv3 [33], MobileNetv2, ResNet-18, ResNet-101 [34], ShuffleNet [35], SqueezeNet [36], and Xception [37].

The differences between these models stem from variations in the structural construction of the connections, parameter optimization, type of connections, designing new block types and operations, width, and depths [38]. GoogLeNet is a small spatial exploitation-based CNN, which examines the surrounding of image pixels (i.e., spatial locality). Thus, the granularity level can be controlled by using filters of the appropriate sizes. This model was the first to introduce splitting, merging, and transformation of features by introducing the inception block. ShuffleNet improved on the computational complexity of GoogLeNet via the process of channel shuffle. The ResNet101(18) and Incpetionv3 models are depth-based CNNs. The premise of such designs is that extremely large number of neuros in one layer can capture the input features with great accuracy, but at the expense of excessive computational cost. On the other hand, a large number of layers would produce the same performance but with greatly reduced overhead. The ResNet introduced the concept of residual learning and used skip connections (i.e., bypass pathways) without incurring more data or parameters. The Inceptionv3 model used small asymmetric filers to improve the computational cost over large symmetric filters. The DenseNet-201, as the name suggests, is a densely-connected network with the goal of solving the vanishing gradient problem with such large number of feed-forward cross-layer connections. The Xception model is width-based multi-connection CNN, which is focused on and utilized the importance of the width of the architecture through parallel processing within the network layers. SqueezeNet uses feature maps and suppresses the less important features using the SE-block, which performs two operations: squeeze (i.e., suppress) and excitation. The MobileNetv2 model was designed for resource-limited mobile devices. This was accomplished by reducing the number of trainable parameters using the depth-wise separable convolution operations. Table 1 shows the number of parameters as calculated from the pre-trained models, the depth of each model in layers, and the required input size.

### 2.3. Performance Evaluation Setup

For a fair comparison, the training parameters were set to the same values for all models. The Number of epochs was set to 10. It was possible to set the minimum batch size to 16 based on the available system memory. The learning rate was 0.003, and the stochastic gradient descent with momentum (SGDM) was used in network training [39].

The dataset was split using three separate different ratios (i.e., 50/50, 70/30, and 90/10). The larger training sets will provide better learning for the CNN models. Augmentation was performed to introduce variety into the dataset. However, the size of the dataset did not increase as only the augmented images were used and the originals were discarded. The augmentations operations were: 1. scaling in the range [0.9, 1.1]; 2. X-Y translation using range [−30, 30] pixels; 3. Random reflection over the horizontal access was performed.

The models were implemented and evaluated using MATLAB R2021a software running on an HP OMEN 30L desktop GT13 with 64 GB RAM, NVIDIA^®^ GeForce RTX^TM^ 3080 GPU, Intel^®^ Core^TM^ i7-10700K CPU @ 3.80 GHz, and 1TB SSD.

### 2.4. Performance Evaluation Metrics

The metrics used to evaluate the performance of the CNN models are shown in Equations (1)–(5). In these equations, $T_{P}$ represent the true positive (i.e., a leaf image correctly classified in one of the nine disease states), $F_{N}$ represent the false negative (i.e., a leaf image classified as healthy but in reality it is drawn from one of the disease classes), $F_{P}$ represent the false positive (i.e., a healthy leaf image wrongly classified as representing a disease), and $T_{N}$ represent the true negative (i.e., a healthy image classified correctly as such). Recall (i.e., true positive rate (TPR) or sensitivity) measures the ability of the model to identify a leaf image as belonging to the correct disease class out of all the positive images, which is affected by the existence of false negatives. Moreover, Specificity measures the ability of the model to identify a leaf image as belonging to the healthy class, which is affected by the existence of false positives. High sensitivity indicates that the model easily recognizes leaf images as representing a disease, but may include a large number of false positives. Precision measures the ratio of false positives to all cases identified as positive (i.e., false positives included). The Accuracy measures the ratio of the sum of true positives and true negatives to the total number of testing images. However, since different classes have different number of images (i.e., class imbalance), the F1 score is considered a more reliable measure of the model classification performance [40].

(1) $$Accuracy=\frac{T_{P}+T_{N}}{P+N}$$

(2) $$Precision=\frac{T_{P}}{T_{P}+F_{P}}$$

(3) $$Recall=\frac{T_{P}}{T_{P}+F_{N}}$$

(4) $$Specificity=\frac{T_{N}}{T_{N}+F_{P}}$$

(5) $$F1=2\times \frac{T_{P}}{2\times T_{P}+F_{P}+F_{N}}$$

## 3. Results and Discussion

Different deep learning models may achieve varying classification performance, require a wide range of training/validation times, or favor certain classes at the expense of others. Moreover, some models may benefit from more training data, while others may be unable to generalize to new data. Furthermore, the performance evaluation needs to account for random choices for the images chosen to be in each subset (e.g., training). Repeating the experiments until favourable results are produced will not provide a faithful reflection of the actual performance. In addition, AI models may be susceptible to overfitting/underfitting. It should be noted that, although fine-tuning certain parameters may improve the results, no such experimentation was performed and standard recommendations from the Matlab R2021a software libraries were followed.

The dataset was split into three different setups: 50/50, 70/30, and 90/10. This will reveal if complex models may be able to learn better from more data in comparison to the smaller ones. In addition, it will help reveal any overfitting or underfitting models. Table 2 displays the average overall F1 score, precision, recall, specificity, and accuracy using 50% of the data for training in addition to the standard deviation (SD) of the accuracy. The results represent the average of 10 runs. The performance of the models was comparably close (i.e., 1.6% difference in F1 score between the best and worst performing models). The highest F1 score and accuracy were 96.5% and 97.5%, respectively. These were achieved by the Inceptionv3 and ResNet101 models.

**Figure 3:**
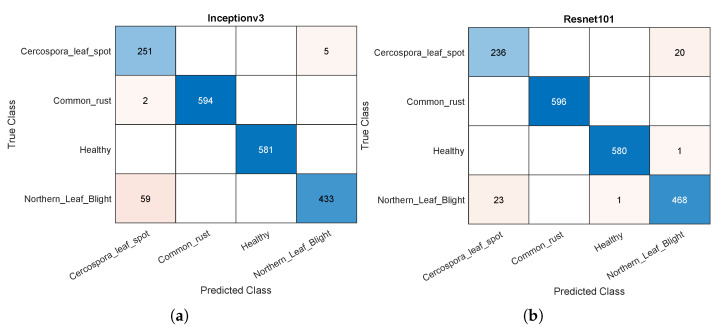
Sample confusion matrices for the Inceptionv3 and ResNet101 models using 50% of the data for training: (**a**) Inceptionv3. (**b**) ResNet101.

**Figure 4:**
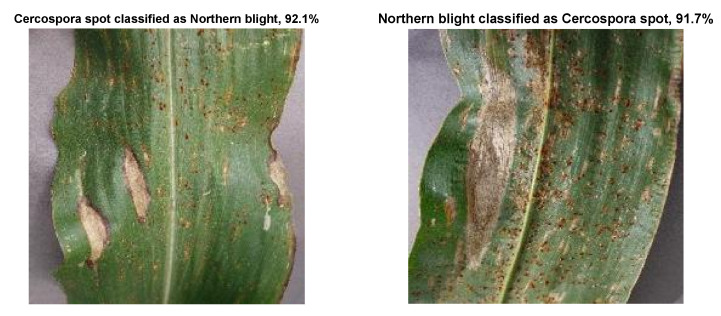
A sample of wrongly classified images along with the class probability.

Further insight into the results is provided by the sample confusion matrices of the Inceptionv3 and ResNet101 models shown in Figure 3. This shows that the major source of errors in Inceptionv3 is caused by classifying some of the images displaying northern leaf blight as Cercospora leaf spot. ResNet101 behaved somewhat similarly, but the misclassification was in both ways for the two aforementioned disease classes. Such errors may be explained by the sample of wrongly classified images shown in Figure 4. To the untrained eye, these two images seem to represent the same disease. However, the dataset provides them in different disease classes. This may be an error in the dataset but only professional pathologists can confirm the gold standard. In this work, the classification in the public dataset was followe.

Increasing the size of the training data subset provide results that corroborate and confirm the good performance that was achieved. Table 3 and Table 4 show the mean overall F1 score, precision, recall, specificity, and accuracy for all models using 70% and 90% of the data for testing, respectively. The Inceptionv3 could not keep up with the lead. Instead, the DarkNet-53 benefitted better from more training data and achieved an F1 score of 97.5% using 70% of the data for training, and 98.0% using 90% of the data of training. The corresponding accuracy values were 98.2% and 98.6%, respectively. Moreover, with an increase size of the training dataset, it exhibited less fluctuation in the accuracy over different runs.

**Figure 5:**
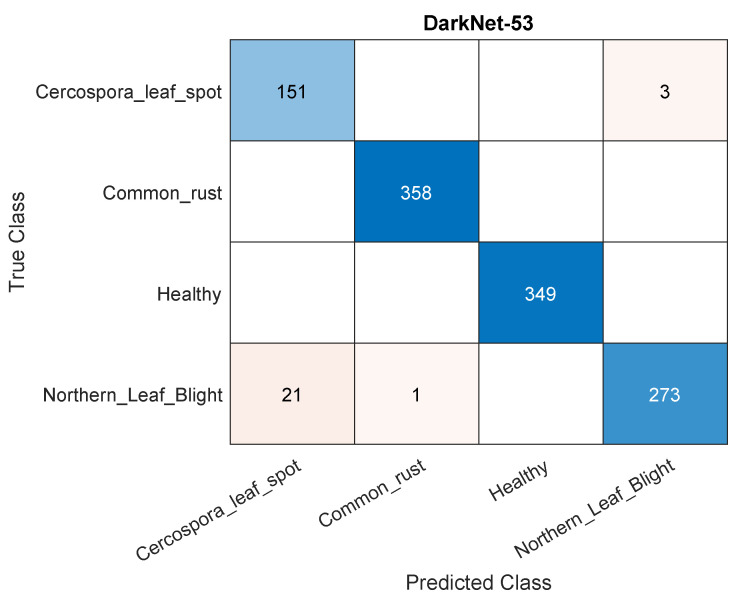
A sample confusion matrix for the DarkNet-53 model and using 70% of the data for training.

**Figure 6:**
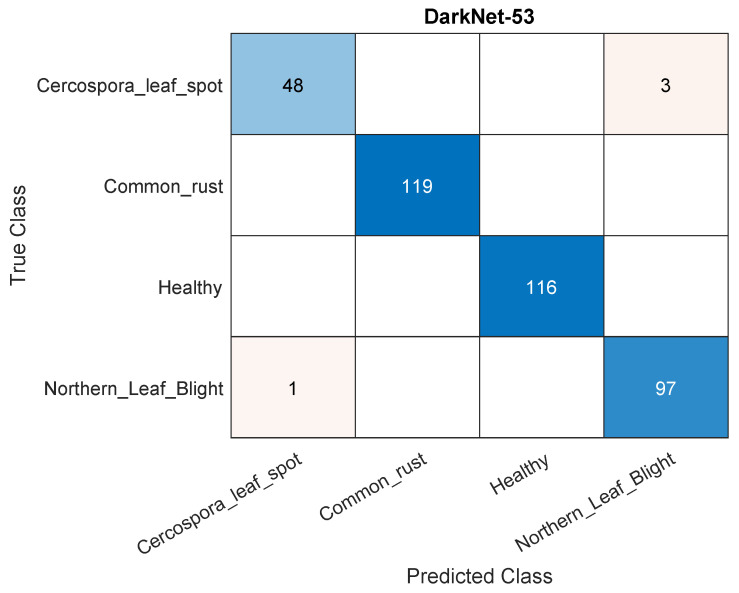
A sample confusion matrix for the DarkNet-53 model and using 90% of the data for training.

**Figure 7:**
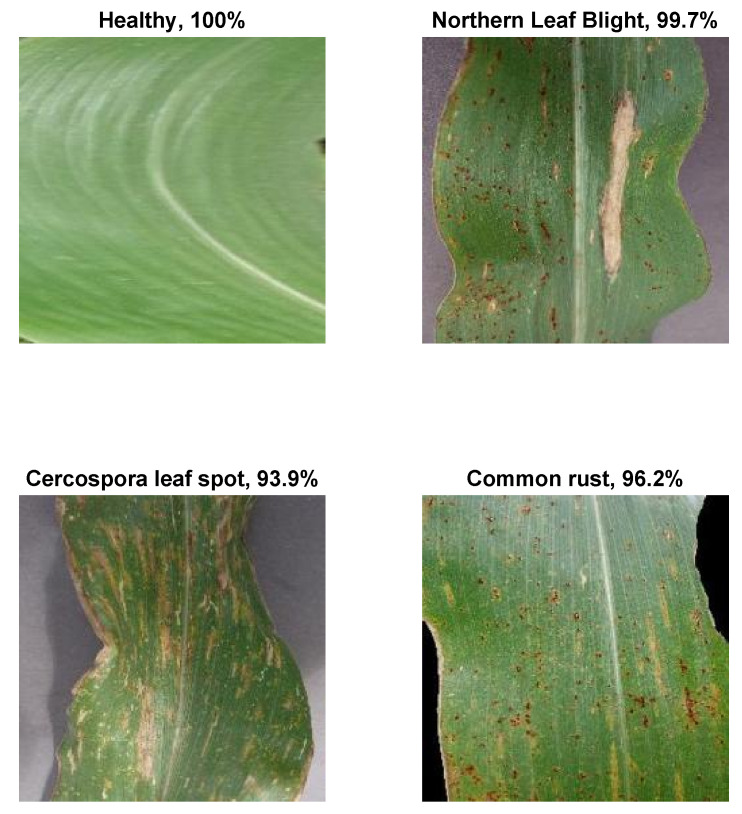
A sample of correctly classified images along with the class probability.

A similar source of classification errors persists when increasing the size of the training data from 50% to 70%. Figure 5 shows a sample confusion matrix for the DarkNet-53 model using 70% of the data for training. Again, the major source of errors is caused by classifying northern leaf blight as Cercospora leaf spot. On the other hand, increasing the training data to 90% of the dataset seemed to reduce such misclassification. This is displayed in Figure 6, which displays a sample confusion matrix for DarkNet-53 and using 90% of the data for training. Much of the errors are rectified, but those remaining are still caused by the northern leaf blight–Cercospora leaf spot misclassification and vice versa. Moreover, the lesser absolute number of errors may be caused by having fewer testing data (i.e., 10% as opposed to 30%), but this is perhaps ruled out as the accuracies in the matrices are within 1% of each other. In other words, relative number show a better performance with more training data. Figure 7 shows samples of correctly classified images along with the corresponding class probability.

Table 5 shows the mean training and validation times for all the models and data split strategies. The table shows roughly three tiers of training/validation speeds: the fast tier comprised of SqueezeNet, GoogLeNet and ResNet18, the very slow tier of DenseNet-201 and Xceptionv, and the medium speed tier containing Inceptionv3, MobileNetv2, ResNet101, ShuffleNet and DarkNet-53. ResNet18 model presents the best compromise in terms of training time (152–195.3 s) and classification performance (96.9–97.8% accuracy). It should be noted, however, that training times do not generally affect the ability to deploy the model. This is because training is conducted offline. Once the model is set with the appropriate weights, it performs classification quickly in individual image testing. Moreover, applications wrapping around the model, does not perform any kind of update to the classification model during deployment. Table 6 shows the inference times per image in milliseconds. The ResNet18 is the model with the fastest inference and represents a good compromise in terms of speed versus accuracy.

The results show that there does not seem any correlation between the input size and having high accuracy in comparison to other models. The models with the largest input size did not achieve the best accuracy, nor they had the longest training/run time. For example, Xception had the longest training time, but inception had somewhat average training time. DenseNet-201 had the highest inference times and DarkNet-53 produced the best accuracy. Moreover, the inference times do not seem to be correlated with the input size.

The agricultural discipline holds a huge potential for great innovation using artificial intelligence. In the context of this work, image-based object detection and classification applications based on deep learning are promising to revolutionize many avenues of research. Nonetheless, several studies have recently been conducted in classifying corn diseases. Padilla et al. [21] developed a system using Raspberry Pi as the hardware platform. Using OpenMP for faster performance, they showed that it is possible to deploy CNN-based applications in practice using limited hardware Amin et al. [23] used different fusion techniques to exploit the synergy of two CNN models: EfficientNetB0 and DenseNet121. However, such an approach may add to the already high computational and memory requirements of deep learning algorithms. Xu et al. [24] and Panigrahi et al. [22] designed their own CNN, with the former modifying AlexNet and the later building a new network from scratch. Although excellent performance was reported, it requires extensive and multiple separate research studies to confirm the validity and performance of new designs in comparison to well-established models. Subramanian et al. [10] used transfer learning with VGG16, ResNet50, InceptionV3, and Xception models, and reported an accuracy range of 83.92% to 99.9% using a dataset of 18,888 images. Table 7 shows a summary of the related literature to identify and classify corn disease.

## 4. Conclusions

Corn is a staple agricultural product that is important in feeding hundreds of millions of people worldwide through largescale as well as hobby farming. Moreover, it forms the basis for many industrial products and biofuels. However, climate change and its consequences in terms of drought, extreme weather conditions, and unseasonable temperatures have put huge strains on the global agricultural output. Furthermore, plant diseases can cause devastation to corn yield and significant financial losses. These factors make it more pressing to deploy technological innovations at various farming scales to protect plants and aid farmers in disease identification and control. In this work, we aimed at using deep learning artificial intelligence algorithms to classify common corn diseases from leaf images. Transfer learning from well-designed and well-evaluated convolutional network models was used to identify the type of disease out of four possible cases (i.e., three diseases and one healthy state). The performance evaluation results show great promise in developing and deploying commercial applications that satisfy the accuracy and ease-of-use requirements. Such efforts will go a long way in helping farmers tackle corn diseases and preserve their livelihood.

The research in this paper can be expanded further by collecting more images of the four classes in the dataset. More data will help develop systems that are more suitable to daily life usage scenarios. For example, in real life, input images may have any type of background, which is not necessarily the same or unified as in the current dataset. Furthermore, more types of corn diseases can be included (e.g., Stewart’s Bacterial Wilt). Moreover, if athe application is ready for field deployment, then it would be useful to incrementally update the deep learning models to increase accuracy and reliability, and counter the phenomena of catastrophic forgetting, which is suffered by such models.

## Figures and Tables

**Table 1:** The depth in terms of number of layers, the number of parameters, and the required input size for each model.

Model	Depth (Layers)	Parameters Count	Input Size
SqueezeNet	18	1,235,496	227×227×3
GoogLeNet	22	6,998,552	224×224×3
Inceptionv3	48	23,851,784	299×299×3
DenseNet-201	201	20,030,952	224×224×3
MobileNetv2	53	35,21,928	224×224×3
ResNet101	101	44,601,832	224×224×3
ResNet18	18	11,694,312	224×224×3
Xception	71	22,906,264	299×299×3
ShuffleNet	162	1,406,712	224×224×3
DarkNet-53	53	41,627,784	256×256×3

**Table 2:** The mean overall F1 score, precision, recall, specificity, and accuracy using 50% of the data for training. The results are an average of 10 runs.

Model	F1 Score	Precision	Recall	Specificity	Accuracy ± SD
SqueezeNet	94.6%	95.0%	94.9%	98.9%	96.4% ± 0.4%
GoogLeNet	95.4%	95.9%	95.0%	99.0%	96.7% ± 0.2%
Inceptionv3	96.5%	96.2%	96.9%	99.2%	97.5% ± 0.5%
DenseNet-201	96.1%	95.7%	96.6%	99.1%	97.2% ± 0.4%
MobileNetv2	95.3%	94.9%	95.8%	98.9%	96.5% ± 0.5%
ResNet101	96.5%	96.3%	96.7%	99.2%	97.5% ± 0.2%
ResNet18	95.6%	95.5%	95.9%	99.0%	96.9% ± 0.4%
Xception	95.6%	96.2%	95.2%	99.0%	97.0% ± 0.3%
ShuffleNet	95.5%	95.1%	96.2%	99.0%	96.7% ± 0.3%
DarkNet-53	96.2%	96.2%	96.5%	99.2%	97.3% ± 0.8%

**Table 3:** The mean overall F1 score, precision, recall, specificity, and accuracy using 70% of the data for training. The results are an average of 10 runs.

Model	F1 Score	Precision	Recall	Specificity	Accuracy ± SD
SqueezeNet	94.9%	95.3%	94.9%	98.8%	96.0% ± 1.3%
GoogLeNet	96.4%	96.6%	96.3%	99.2%	97.4% ± 0.2%
Inceptionv3	96.9%	96.8%	97.2%	99.3%	97.8% ± 0.5%
DenseNet-201	97.4%	97.1%	97.8%	99.4%	98.1% ± 0.1%
MobileNetv2	96.8%	96.6%	97.1%	99.3%	97.7% ± 0.5%
ResNet101	97.1%	96.7%	97.7%	99.4%	97.9% ± 0.4%
ResNet18	96.9%	96.8%	97.0%	99.3%	97.8% ± 0.4%
Xception	96.9%	97.0%	96.8%	99.3%	97.8% ± 0.2%
ShuffleNet	96.8%	96.5%	97.2%	99.3%	97.7% ± 0.2%
DarkNet-53	97.5%	97.3%	97.8%	99.4%	98.2% ± 0.5%

**Table 4:** The mean overall F1 score, precision, recall, specificity, and accuracy using 90% of the data for training. The results are an average of 10 runs.

Model	F1 Score	Precision	Recall	Specificity	Accuracy ± SD
SqueezeNet	95.9%	96.0%	96.0%	99.1%	97.1% ± 0.7%
GoogLeNet	96.4%	96.5%	96.5%	99.2%	97.5% ± 0.7%
Inceptionv3	97.0%	96.7%	97.5%	99.4%	97.9% ± 0.3%
DenseNet-201	97.2%	96.8%	97.6%	99.4%	98.0% ± 0.4%
MobileNetv2	96.1%	95.8%	96.7%	99.2%	97.2% ± 0.9%
ResNet101	97.3%	97.1%	97.6%	99.4%	98.0% ± 0.8%
ResNet18	96.3%	96.2%	96.6%	99.2%	97.4% ± 0.7%
Xception	95.7%	95.4%	96.0%	99.0%	96.9% ± 0.5%
ShuffleNet	96.5%	96.4%	96.7%	99.2%	97.6% ± 0.4%
DarkNet-53	98.0%	97.9%	98.1%	99.6%	98.6% ± 0.5%

**Table 5:** The mean training and validation times for all algorithms and all data split strategies. All times are in seconds.

Data Split	50/50	70/30	90/10
Model			
SqueezeNet	146.0	159.9	176.9
GoogLeNet	244.3	295.6	353.3
Inceptionv3	698.3	871.5	1057.8
DenseNet-201	2257.7	2789.5	3376.3
MobileNetv2	960.8	1244.0	1544.6
ResNet101	684.33	866.2	1059.9
ResNet18	152.0	173.7	195.3
Xception	2633.0	3464.3	4221.9
ShuffleNet	716.6	941.9	1141.6
DarkNet-53	565.8	710.2	840.2

**Table 6:** The mean ± SD inference times per image for all algorithms. All times are in milliseconds.

Model	Inference Time Per Image (ms)
SqueezeNet	1.267 ± 0.034
GoogLeNet	1.313± 0.031
Inceptionv3	4.76 ± 0.076
DenseNet-201	13.13 ± 0.045
MobileNetv2	2.31 ± 0.058
ResNet101	2.211 ± 0.07
ResNet18	0.724 ± 0.011
Xception	7.4 ± 0.056
ShuffleNet	1.304 ± 0.009
DarkNet-53	6.944 ± 0.063

**Table 7:** A summary of the related literature to identify and classify corn diseases.

Study	Objective	Dataset	Approach	Performance
Padilla et al. [21]	Classification (three diseases + one healthy + one unknown)	-	OpenMP implementation of a CNN on Raspberry Pi	Highest accuracy = 93%
Amin et al. [23]	Classification (three diseases + one healthy)	15,408 leaf images	Fusion of EfficientNetB0 and DenseNet121	Accuracy = 98.56%
Xu et al. [24]	Classification (three diseases + one healthy)	17,600 augmented leaf images	Modified AlexNet	Accuracy = 93.28%
Panigrahi et al. [22]	Classification (three diseases + one healthy)	3823 leaf images	Custom nine-layer CNN	Accuracy = 98.78%
Subramanian et al. [10]	Classification (three diseases + one healthy)	18,888	VGG16, ResNet50, InceptionV3, and Xception	Accuracy range from 83.92% to 99.9%
This work	Classification (three diseases + one healthy)	3852 leaf images	Ten CNN models	Accuracy = 98.6%

## Data Availability

Not applicable.

## Abbreviations

The following abbreviations are used in this manuscript:

AI	artificial intelligence
ANN	artificial neural networks
MLP	multilayer perceptron
CNN	Convolutional neural network
SVM	Support vector machine
$T_{P}$	True positive
$T_{N}$	True negative
$F_{N}$	False negative
$F_{P}$	False positive
ReLU	Rectified linear unit
TPR	True positive rate
N	Negatives
P	Positives
SGDM	Stochastic gradient descent with momentum
SD	Standard deviation
